# A Prediction of NPVR ≥ 80% of Ultrasound-Guided High-Intensity Focused Ultrasound Ablation for Uterine Fibroids

**DOI:** 10.3389/fsurg.2021.663128

**Published:** 2021-06-04

**Authors:** Mei-Jie Yang, Ren-Qiang Yu, Wen-zhi Chen, Jin-Yun Chen, Zhi-Biao Wang

**Affiliations:** ^1^State Key Laboratory of Ultrasound in Medicine and Engineering, College of Biomedical Engineering, Chongqing Medical University, Chongqing, China; ^2^Chongqing Key Laboratory of Biomedical Engineering, Chongqing Medical University, Chongqing, China; ^3^College of Medical Informatics, Chongqing Medical University, Chongqing, China; ^4^Department of Radiology, First Affiliated Hospital of Chongqing Medical University, Chongqing, China; ^5^Ultrasound Ablation Center, First Affiliated Hospital of Chongqing Medical University, Chongqing, China

**Keywords:** high-intensity focused ultrasound, ultrasound ablation, uterine fibroids, NPVR, patient screening

## Abstract

**Objective:** To evaluate factors in predicting the treatment outcome of ultrasound-guided high-intensity focused ultrasound (USgHIFU) ablation for uterine fibroids with a non-perfused volume ratio (NPVR) of at least 80%.

**Methods:** One thousand patients with uterine fibroids who received USgHIFU were enrolled. Thirty-two independent variables of four dimensions of data set, including general information of patients, clinical symptoms, laboratory tests, and fibroid imaging characteristics, were used to investigate the potential predictors of the NPVR of at least 80% by multivariate logistic regression. NPVR was the gold standard for evaluating the efficiency of HIFU ablation, and a NPVR of at least 80% was considered sufficient ablation, while partial ablation was defined as having an NPVR of <80%.

**Results:** Out of 1,000 fibroids, 758 obtained sufficient ablation and 242 obtained partial ablation, and the median NPVR was 88.3% (interquartile range: 80.3–94.8%). The probability of NPVR reaching 80% fibroids with a signal intensity of T2WI of hypointense, isointense, and hyperintense was 86.4, 76.5, and 62.6%, respectively; fibroids with an enhancement type of T1WI of slight, irregular, and regular was 81.5, 73.6, and 63.7%, respectively; and fibroids with uterine anteroposterior of 30–130 mm was 57.7–78.3%, respectively. In patients with a platelet count of 50 × 10^9^/L−550 × 10^9^/L, the probability of NPVR reaching 80% is from 53.4 to 80.1%, respectively.

**Conclusions:** In predicting NPVR ≥ 80%, the signal intensity on T2WI was the most important factor affecting ablative efficiency, followed by enhancement type on T1WI, uterine anteroposterior, and platelet count.

## Introduction

Uterine fibroid is the most common benign tumor in women of childbearing age. Up to 25% of women and up to 30–40% of women older than 40 years have clinical symptoms of uterine fibroids (1–3). Many patients show clinical symptoms such as menorrhagia, irregular bleeding, pelvic pain, or infertility (4–6), which have a significant negative effect on their quality of life. Currently, the management for uterine fibroids includes surgery, medicine, uterine artery embolization (UAE), radiofrequency ablation (RFA), ultrasound-guided high-intensity focused ultrasound (USgHIFU), and magnetic resonance-guided high-intensity focused ultrasound (MRgHIFU) (7–11).

HIFU ablation for uterine fibroids (UFS) was to directly focus the ultrasound beam to the tumor to cause coagulative necrosis of the target, thus achieving the purpose of treating uterine fibroids (12, 13). The non-perfused volume ratio (NPVR) of HIFU ablation of uterine fibroids, is the ratio of the volume of the non-perfusion area in the postoperative enhanced MR image to the volume of the fibroids, which is related to symptom relief and can be used as the imaging gold standard for evaluating the effect of HIFU ablation (14, 15). Previous studies have shown that when the NPVR of uterine fibroids is up to 70%, the 2-year clinical effect of ultrasound ablation is considered to be equivalent to myomectomy (16, 17). A NPVR of at least 90%, even almost 100% of the fibroid tumor volume without compromising safety was recommended (18, 19). However, due to the histological characteristics of uterine fibroids and technical limitations, not all uterine fibroids could obtain NPVR of 90–100% (20). Park et al. reported that in USgHIFU ablation of uterine fibroids, achievement of an immediate NPVR of at least 80% is safe, with greater tumor volume shrinkage compared with cases with a lower NPVR (19). A number of studies have shown that the average or median NPVR of USgHIFU ablation of uterine fibroids has reached more than 80% (9, 21, 22). One of the studies was from 20 centers, including 16 inexperienced centers. Therefore, the measure of achieving 80% of NPVR is technically feasible and meets the clinical efficacy (9, 23, 24). Previous studies have focused on assessing the ablation efficacy based on tissue in the acoustic pathways, MRI signal intensity, structure, and blood supply (22, 25). Other clinical information, such as clinical symptom data and laboratory test data of uterine fibroids, was ignored. Fan et al. used multiple linear regression to find that the factors influencing the NPVR of HIFU ablation of uterine fibroids were as follows: distance from UFs ventral side to skin, hypointense and hyperintense signal of T2WI, enhancement type of T1WI, posterior and anterior location of UFs, transmural type of UFs, and anteverted uterine position (26). Lee et al. used multiple regression to find that the T2WI signal intensity of uterine fibroids relative to pelvic muscles was the only important factor related to NPVR (27). Li et al. found that the signal type of T2WI was correlated with NPVR by using the quantitative perfusion parameters of dynamic contrast-enhanced MRI (28). Keserci et al. used MRI parameters to predict the effect of HIFU ablation of uterine fibroids. It is found that in addition to the myoma and muscular layer of T2 signal intensity ratio, the abdominal subcutaneous fat layer thickness, fibroids peak enhancement, and fibroids reaching the peak time could be used to predict whether the NPVR reached 90% (20). Therefore, this study intends to incorporate the multidimensional clinical data obtained from a large sample, to further explore the factors affecting the ablative efficiency of HIFU ablation and analyze the relative importance of its contribution to the efficiency, and that provided a more accurate ablative efficiency prediction and the factors influencing ablative efficiency could optimize patient screening for HIFU ablation of uterine fibroids.

## Materials and Methods

### Patients

The study protocol was approved by the Ethics Committee of the First Affiliated Hospital of Chongqing Medical University. Patients with UFs who received a single-session USgHIFU at the First Affiliated Hospital of Chongqing Medical University from January 2013 to June 2018 were enrolled. Prior to enrollment, the fibroid status and treatment plan were evaluated by a gynecologist, a radiologist, and a HIFU physician. Before HIFU treatment, the details of the treatment were discussed with all patients, who then signed a consent form. This retrospective study was approved by our institutional review board, and informed consent was waived, because the data are anonymized. All procedures followed were in accordance with ethical standards and the Declaration of Helsinki.

The inclusion criteria were as follows: (1) premenopausal patients over 18 years old; (2) in the acoustic pathway, there is no pubic bone, and there is no intestine immovable; (3) patients could communicate with the medical staff during procedure; (4) patients agreed to undergo pre-treatment and post-treatment enhanced MRI scanning; (5) the size of the UFs was between 3 and 11 cm. Only the largest fibroid (i.e., the most symptomatic one) was selected for investigation if the patient had multiple fibroids in this study.

The exclusion criteria were as follows: (1) patients who were contraindicated for MRI scanning or gadolinium-injection solution; (2) patients with significant degenerative fibroids or suspected uterine malignancy assessed by enhanced MRI; (3) special category of fibroids, such as pedunculated subserous or submucosal fibroids; (4) patients with scar tissue in the acoustic pathway, causing obvious attenuation of the B-mode ultrasound behind the detection of tissues (sound attenuation width ≥ 15 mm); (5) patients who were unable to lie in a prone position for 2 h.

### MRI Evaluation

All patients received MRI scanning before and within 1 week after the treatment. A series of T1WI, T2WI, and enhanced T1WI were performed with a 3.0-T MRI system (GE Medical system, Milwaukee, WI, USA).

The volume of fibroids was measured on T2W images to obtain data from these three dimensions: longitudinal diameter (D1), anteroposterior diameter (D2), and transverse diameter (D3). The non-perfused volume (NPV) was evaluated on enhanced T1W images after the treatment. The volume was calculated using the following equation: *V* = 0.5233 × D1 × D2 × D3 (17). The NPV rate was defined as NPV/post-treatment fibroid volume × 100%.

All MR images were evaluated by three experienced radiologists and were recorded as follows: locations of uterus: anteverted, median, retroverted; locations of uterine fibroid: anterior wall, lateral wall, posterior wall, and fundus; types of uterine fibroid: intramural, subserous, and submucous; three-dimensional diameters of fibroid and uterine; distance from ventral side of fibroid to skin: the shortest distance from ventral side to skin of maximal diameter of fibroid; subcutaneous fat layer thickness: the thickness of the subcutaneous fat layer on the inferior border of the second sacral vertebrae according to the T2WI; rectus abdominis thickness: the thickness of the rectus abdominis on the inferior border of the second sacral vertebrae according to the T2WI; distance from center of fibroid to sacrum: the shortest distance from the center of the fibroid to the sacrum of the maximal diameter of the fibroid. The T2WI signal intensity of uterine fibroids was classified according to Funaki into three types: hypointense, isointense, and hyperintense (14). According to the degree of enhancement of uterine fibroids compared to that of the myometrium 60 s after gadolinium injection (29), the enhanced type on TIWI was divided into three types: slight enhancement, regular enhancement, and irregular enhancement.

### Ultrasound-Guided HIFU Ablation

HIFU ablation was performed by HIFU-licensed physicians with at least 3 years of HIFU clinical experience using a model-JC Focused Ultrasound Tumor Therapeutic System (Chongqing Haifu Medical Technology Co., Ltd., Chongqing, China). The equipment combined with an ultrasonic imaging device, which provided real-time guiding during ablation. The experimental parameters in this study include the following: The operating frequency of the US transducer was 0.8 MHz and energy was adjustable in the range 200–400 W. Circulating degassed water was used as the coupling medium and the focal region was 1.5 × 1.5 × 10.0 mm. The patients were placed in a prone position on the HIFU therapy table, with the anterior abdominal wall in contact with degassed water. A catheter was inserted into the bladder and degassed normal saline was filled in set for the purpose of properly filling the bladder. A degassed water balloon was placed between the abdominal wall and transducer to compress and push away the bowel from the acoustic pathway during treatment. Fentanyl-midazolam was used to keep conscious sedation. The ablation results were monitored based on the gray changes in the target area displayed by ultrasound imaging, and the sonication was terminated when the increased gray scale covered the planned ablation area (30, 31). For patients with multiple fibroids, the main fibroid was treated first, and other fibroids were also ablated under the control of total treatment time within 3 h. All the complications were recorded and graded according to the SIR classification standard by the Society of Interventional Radiology In SIR classification, grades C–F are major complications (32, 33).

### Ablation Effect Analysis

According to the NPVR, sufficient ablation was with a NPVR of at least 80% while partial ablation was with a NPVR of <80% (24).

### Statistical Analysis

The data that followed the normal distribution were presented as the mean ± standard deviation. The skewed distribution data were presented as the median and interquartile range. The categorical variables were described by the total number of categories. The chi-square test and Mann–Whitney *U*-test were utilized for univariate analysis. Binary logistic regression analysis was utilized for multivariate analysis. *P* < 0.05 was considered statistically significant. All model and statistical analyses were performed by R 3.5.3 (The R Foundation for Statistical Computing, Vienna, Austria).

## Results

### Patients and Ultrasound Ablation Results

**Figure 1:**
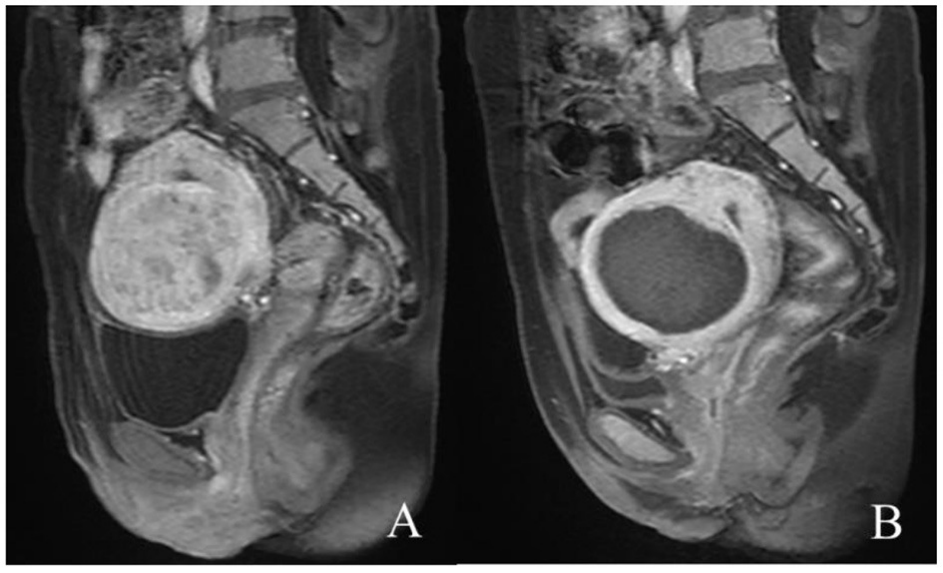
Contrast-enhanced MR images before and after HIFU treatment. **(A)** The fibroid was submucous, anterior, and hyperintense before treatment; **(B)** the non-perfused volume was shown inside uterine fibroid after treatment.

A total of 1,000 cases with 1,000 fibroids were enrolled; mean age was 40 years (range, 35–44 years). The maximum size of the uterine fibroids was 55.5 ± 14.2 mm (range, 30–106 mm), and the volume was 181,089 ± 134,283.1 mm^3^ (range, 24,360–824,784 mm^3^). Out of 1,000 fibroids, 758 obtained sufficient ablation (Figure 1) and 242 obtained partial ablation, and the median NPVR was 88.3% (interquartile range: 80.3–94.8%). The median sonication power of 1,000 fibroids was 400 W (interquartile range: 395–400 W), the median sonication time was 852 s (interquartile range: 544–1,348 s), and the median total dosage was 328,440.0 J (interquartile range: 206,772.5–528,800.0 J) (Table 1).

**Table 1:** Baseline characteristics and the results of ultrasound ablation.

**Parameter**	**Data**
Age (years)^*^	40 (35–44)
Size of uterine fibroids (mm)	55.5 ± 14.2 (30–106)
Volume of uterine fibroids (mm^3^)	181,089 ± 134,283.1 (24,360–824,784)
Ultrasonic power (W)^*^	400 (395–400)
Sonication time (sec)^*^	852 (544–1,348)
Total dose (J)^*^	328,440.0 (206,772.5–528,800.0)
NPV (mm^3^)^*^	75,837 (36,196–96,232)
NPVR (%)^*^	88.3% (80.3–94.8%)

*NPV, non-perfused volume; NPVR, non-perfused volume ratio*.

* *Data are median (interquartile range)*.

### Comparison of the Characteristics in Patients' Sufficient Ablation and Partial Ablation

The 758 patients with NPVR ≥ 80% of uterine fibroids were in the sufficient ablation group, while 242 were in the partial ablation group. The characteristic data of patients with uterine fibroids were grouped into four dimensions of data set: general information of patients, clinical symptoms, laboratory test, and fibroid imaging characteristics. The characteristics were compared between the sufficient ablation group and the partial ablation group. Univariate analysis showed platelet count, uterine anteroposterior, type of uterine fibroids, location of uterine fibroids, signal intensity on T2WI, and enhancement type on T1WI as the significant factors between the two groups (*P* < 0.05) (Table 2).

**Table 2:** Comparison of characteristics between two groups.

**Variable**	**Sufficient ablation**	**Partial ablation**	***P*-value**
**General patient data**			
Age (years)^*^	40.0 (35.0–44.0)	40.0 (34.0–44.0)	0.302
Height (cm)^*^	158.0 (155.0–160.0)	158.0 (155.0–160.0)	0.675
BMI (kg/m^2^)^*^	22.2 (20.6–24.0)	22.2 (20.5–24.2)	0.617
Thickness of rectus abdominis (mm)^*^	9.1 (7.6–11.0)	9.0 (7.3–11.0)	0.412
Thickness of subcutaneous fat layer (mm)^*^	16.0 (12.1–21.1)	16.6 (12.8–22.0)	0.944
Scar in the lower abdomen (no/yes) (*n*)	181/577	61/181	0.739
**Clinical symptom data (no/mild/moderate/severe/serious)**			
Increased menstruation (*n*)	349/181/139/66/23	114/59/50/18/1	0.431
Blood clot (*n*)	193/352/148/47/18	60/109/56/16/1	0.282
Prolonged menstrual period (*n*)	429/162/116/41/10	132/56/40/14	0.431
Irregular menstrual cycle (*n*)	413/223/90/24/8	133/70/23/13/3	0.493
Lower abdominal discomfort (*n*)	257/360/100/36/5	91/102/38/9/2	0.525
Frequent urination during the day (*n*)	493/193/51/18/3	163/50/21/5/3	0.276
Frequent urination at night (*n*)	510/180/49/15/4	170/55/15/1/1	0.525
Fatigue (*n*)	394/227/100/30/7	132/78/26/5/1	0.551
**Laboratory test data**			
Hemoglobins (g/L)^*^	128.0 (113.0–137.0)	128.0 (117.5–137.8)	0.421
Leukocyte count (10^9^/L)^*^	5.5 (4.6–6.5)	5.6 (4.6–6.6)	0.598
Lymphocyte absolute value (10^9^/L)^*^	1.6 (1.3–2.0)	1.6 (1.3–2.0)	0.884
Monocyte absolute value (10^9^/L)^*^	0.3 (0.3–0.4)	0.3 (0.3–0.4)	0.621
Platelet count (10^9^/L)^*^	235.0 (193.2–285.8)	228.0 (186.0–265.8)	0.035^*^
Red blood cell count (10^12^/L)^*^	4.4 (4.1–4.6)	4.4 (4.1–4.6)	0.790
**MRI examination**			
Uterine transverse (mm)^*^	72.3 (63.0–84.0)	71.8 (61.0–82.0)	0.137
Uterine anteroposterior (mm)^*^	73.0 (60.0–84.0)	67.7 (57.0–81.7)	0.014^*^
Uterine longitudinal (mm)^*^	93.0 (81.0–104.0)	92.0 (80.3–102.0)	0.477
Fibroid transverse (mm)^*^	51.0 (43.0–61.4)	51.0 (43.0–62.0)	0.648
Fibroid anteroposterior (mm)^*^	51.0 (42.6–61.0)	51.0 (43.0–60.0)	0.916
Fibroid longitudinal (mm)^*^	53.0 (45.0–64.0)	54.0 (45.0–56.0)	0.593
Type of uterine fibroids (submucous/subserous/intramural) (*n*)	134/122/502	31/63/148	0.001^*^
Location of uterine (anteverted/median/retroverted) (*n*)	476/76/206	159/21/62	0.686
Location of uterine fibroids (anterior/posterior/lateral/fundus) (*n*)	321/183/204/50	79/80/71/12	0.011^*^
Signal intensity on T2WI (hypointense/isointense/hyperintense) (*n*)	268/325/165	54/73/115	<0.00^*^
Enhancement type on T1WI (slight/irregular/regular) (*n*)	483/115/160	111/38/93	<0.00^*^
Distance from center of fibroid to sacrum (mm)^*^	48.1 (38.8–61.7)	46.2 (37.3–59.3)	0.150
Distance from ventral side of fibroid to skin (mm)^*^	39.0 (28.3–53.9)	42.0 (28.9–61.2)	0.106

* *Data are median (interquartile range); BMI, body mass index*.

### Evaluation of the Factors Affecting Ablative Efficiency

The multivariate logistic regression analysis showed that platelet count, uterine anteroposterior, signal intensity on T2WI, and enhancement type on T1WI were all independent risk factors for ablative efficiency (*P* < 0.05). The patient with a larger size of uterine had a greater risk of sufficient ablation than patients with a smaller size of uterine; the patient with higher platelet count had greater risk of sufficient ablation than patients with lower platelet count; the patient with hypointense T2WI signal of uterine fibroid had greater risk of sufficient ablation than patients with isointense and hyperintense T2WI signal of uterine fibroid; the patient with slight enhancement T1WI of uterine fibroid had greater risk of sufficient ablation than patients with irregular and regular enhancement T1WI of uterine fibroid (Table 3).

**Table 3:** The binary logistic regression analysis of variance.

	**Estimate**	**Std. error**	***z*-value**	**OR1 (95% CI)**	***P*-value**	**Standardized coefficient**
(Intercept)	1.504	0.448	3.357	4.501 (1.875, 10.883)	<0.000	
Uterine anteroposterior	0.013	0.005	2.795	1.013 (1.004, 1.022)	0.005	0.130
Platelet count	0.003	0.001	2.615	1.003 (1.001, 1.005)	0.009	0.114
Signal intensity on T2WI	−0.658	0.105	−6.232	0.518 (0.420, 0.636)	<0.000	−0.281
Enhancement type on T1WI	−0.368	0.088	−4.188	0.692 (0.582, 0.822)	<0.000	−0.174

### The Degree of Importance of Ablative Efficiency-Influencing Factors

**Figure 2:**
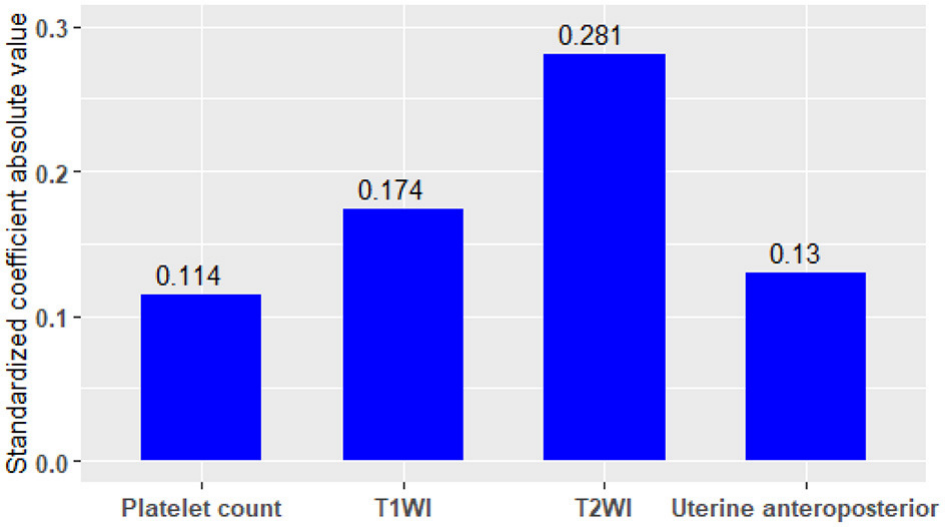
Comparison of the degree among the factors influencing ablative efficiency.

Compared to the absolute value of standardized coefficients (34), the most important factor affecting ablative efficiency was signal intensity on T2WI, followed by enhancement type on T1WI, uterine anteroposterior, and platelet count (Figure 2).

### Logistic Regression Analysis of NPVR ≥ 80%

**Figure 3:**
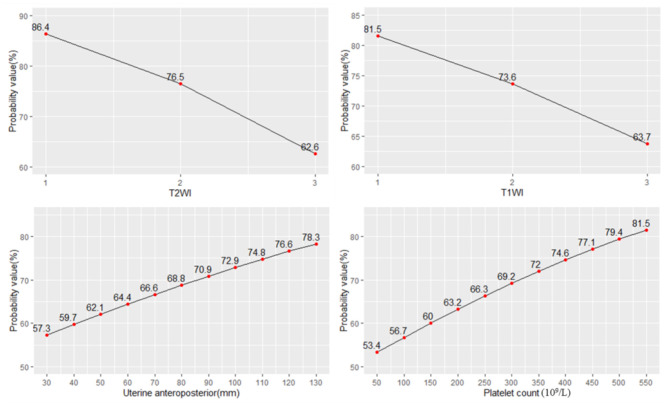
The probability of NPVR reaching 80% based on the four most important independent factors.

The probability of NPVR reaching 80% with signal intensity of T2WI of hypointense, isointense, and hyperintense was 86.4, 76.5, and 62.6%, respectively. The probability of NPVR reaching 80% with enhancement type of T1WI of slight, irregular, and regular was 81.5, 73.6, and 63.7%, respectively. The probability of NPVR reaching 80% with uterine anteroposterior of 30, 40, 50, 60, 70, 80, 90, 100, 110, 120, and 130 mm was 57.7, 59.7, 62.1, 64.4, 66.6, 68.8, 70.9, 72.9, 74.8, 76.6, and 78.3%, respectively. The probability of NPVR reaching 80% with a platelet count of 50 × 10^9^/L, 100 × 10^9^/L, 150 × 10^9^/L, 200 × 10^9^/L, 250 × 10^9^/L, 300 × 10^9^/L, 350 × 10^9^/L, 400 × 10^9^/L, 450 × 10^9^/L, 500 × 10^9^/L, and 550 × 10^9^/L was 53.4, 56.7, 60.0, 63.2, 66.3, 69.2, 72.0, 74.6, 77.1, 79.4, and 80.1%, respectively (Figure 3).

## Discussion

Focused ultrasound ablation surgery for uterine fibroids, due to its results in rapid recovery and low risks of complications, would probably provide effective management until menopause (35). Ultrasound and MRI are two guidance modalities integrated with HIFU, which is an essential prerequisite of HIFU treatment for patient selection, treatment planning, real-time treatment monitoring, safe delivery of the desired therapeutic dose, and treatment efficacy evaluation. Most HIFU treatments are still performed under ultrasound imaging guidance mainly due to its cost-effectiveness and greater accessibility to patients (10). The purpose of HIFU ablation of uterine fibroids is to cause coagulation necrosis of target fibroids. The NPVR, as a measure of technical success, is recognized as a predictor of clinical outcome for HIFU ablation of fibroids. Previous studies suggest that the factors affecting NPVR include tissue characteristics of the uterine fibroids and technical limitations in HIFU treatment. Tissue characteristics are the T1 and T2 signal intensity (SI), and fibroid type defined by general uterine position, fibroid size, and number; technical limitations in HIFU treatment are presence of scar tissue, excessive abdominal subcutaneous fat, distance between the skin and the fibroids, distance between the sacral bone surface and the fibroids, and bowel in the path of sonication (13).

The influential factors of complex clinical characteristics data in the efficacy of HIFU ablation are of great significance for accurate preoperative screening of patients and prediction of efficacy.

Multivariate logistic regression study found that among 32 variables in four dimensions, the four most important independent factors influencing NPVR were, in order of importance, signal intensity on T2WI, enhanced type onT1WI, uterine anteroposterior, and platelet count.

The signal intensity on T2WI was the most important indicator influencing the efficiency of ultrasound ablation, and the higher the signal intensity of T2WI was, the worse the efficiency of ablation would be. According to the imaging pathology results, the histological features of hypointensity fibroids were as follows: the cells in the fibroid are closely arranged with less liquid and mucoid and more collagen fibers, making it easier to ablate, whereas T2 hyperintensity fibroids contain more cellular components and fewer collagen fibers, which are not conducive to energy deposition (24). The regression analysis showed that the hypointensity fibroid ablation rate reached 80% with the probability of 86.4%; isointensity, 76.5%; and hyperintensity, 62.6%. The sufficient ablation probability of hypointensity and isointense T2WI signal was greater than that of hyperintense T2WI signal. In clinical screening cases, fibroids with hyperintense T2WI signal should be considered as the most important exclusion factor, and the possibility that cannot achieve the ideal NPVR should be evaluated.

Dynamic contrast-enhanced MRI T1 perfusion-based classification is a strong predictor of treatment outcome (24, 36). T1 perfusion-based classification had a very strong correlation with immediate NPVR and the mean immediate NPVR in slight and irregular fibroids was higher than in regular fibroids (36). Dynamic contrast-enhanced MRI showed that the ablative efficiency of regular enhancement fibroids was not sufficient compared with slight and irregular enhanced fibroids. Dynamic contrast-enhanced MRI showed that the ablative efficiency of regular enhancement fibroids was not sufficient compared with slight and irregular enhanced fibroids. The blood perfusion might be the main parameter affecting temperature rise, which, in turn, affects the ablation efficacy (36). Dynamic enhanced MRI reflects the blood supply of uterine fibroids: regular enhanced fibroids are rich in blood supply, slightly enhanced fibroids indicate a lack of blood supply, and irregular enhanced fibroids suggest that ischemia or teratogenic necrosis may exist in the fibroids. When uterine fibroids are ablated by ultrasound, the rich blood flow removes heat so that the energy is not easily deposited, leading to difficult ablation. It is easier to ablate fibroids with a lack of blood or denatured necrosis (24). The regression analysis showed that the slight enhanced fibroid ablation rate reached 80% with the probability of 81.6%; irregular enhancement, 73.6%; and regular enhancement, 63.7%. Therefore, the enhancement degree of dynamic enhanced MRI uterine fibroids should be regarded as one of the important basis for screening cases, especially the fibroids with hyperintense intensity on T2WI, which can be excluded in clinical practice.

The uterine anteroposterior reflected the size of uterine fibroids. The probability of sufficient ablation was higher with the increase of uterine fibroids in a certain range. First, UFs had an expansive growth pattern of benign tumors, and with the growth of fibroids, especially above 7 cm, the internal echo of fibroids became heterogeneous during ultrasound scanning, and the inhomogeneous acoustic tissue properties would cause an increase in the scattering and absorption of the acoustic wave. Second, when the diameter of the fibroids increased above 5 cm, the blood supply inside the fibroids was relatively insufficient, which was the main reason for the degeneration of fibroids. However, due to the presence of volumetric effects, enhanced MRI did not show perfusion defects, and ultrasonic energy was easily deposited in tissues with relatively insufficient blood supply and degeneration. Third, the vascular effect of ultrasound caused vascular necrosis and blood flow blockage, which led to a reduction in ultrasound dose for subsequent tissue ablation. For larger tumors, the thermal injury spread locally to untreated areas, and which resulted in the NPV being larger than the planned treatment area (37–39). The results of logistic regression showed that the probability of NPVR reaching 80% with uterine anteroposterior of 30, 40, 50, 60, 70, 80, 90, 100, 110, 120, and 130 mm was 57.7, 59.7, 62.1, 64.4, 66.6, 68.8, 70.9, 72.9, 74.8, 76.6, and 78.3%, respectively. Therefore, for larger fibroids, multiple factors led to the range of injury and NPVR increasing. However, the enlargement of the uterus to or above the umbilical level was not conducive to sufficient ablation, which can be treated by fractional ablation or multiple clinical strategies. For multiple fibroids, the long-term ablative efficiency may be affected by the clinical recurrence because of the growth of residual fibroids. Considering the repeatability of HIFU ablation, effective ablation for visible fibroids was still an alternative treatment for such fibroids.

This study first found that the peripheral blood platelet count was associated with the ablative efficiency. Previous studies had shown that platelet count-derived growth factor (PDGF) and PDGF-R were normally expressed in myometrium smooth muscle cells and uterine fibroids. PDGF-R sites in leiomyoma cells were more than those in myometrium cells. The sites of PDGF-AA, PDGF-BB, PDGF-CC, and PDGF-DD were higher expressed in myoma than in myometrium (40, 41). Liu found that platelet count was related to the formation and development of adenomyosis, and the increase of platelet count may promote tissue fibrosis (42). Therefore, whether the increase of platelet count in peripheral blood also increased the microenvironment of uterine fibroids and changed the acoustic environment of the tissues, thus affecting the deposition of ultrasonic energy and further affecting the ablative efficiency, needs to be further studied. The probability of NPVR reaching 80% with a platelet count of 50 × 10^9^/L, 100 × 10^9^/L, 150 × 10^9^/L, 200 × 10^9^/L, 250 × 10^9^/L, 300 × 10^9^/L, 350 × 10^9^/L, 400 × 10^9^/L, 450 × 10^9^/L, 500 × 10^9^/L, and 550 × 10^9^/L was 53.4, 56.7, 60.0, 63.2, 66.3, 69.2, 72.0, 74.6, 77.1, 79.4, and 80.1%, respectively. Peripheral platelet count can also be used as a predictor of sufficient ablation.

The limitation of this study is the unique characteristics of this patient cohort. These patients had a thin subcutaneous fat layer of average 16 mm (which may be common in China/Asia). However, this is not the case in other parts of the world where patients are usually more obese. Therefore, the predictive efficacy of other demographic characteristics needs to be further verified, and long-term follow-up results still need to be paid attention to.

## Conclusion

Considering the complexity of clinical data, the inclusion of multidimensional data facilitates the prediction of ablative efficiency. Signal intensity on T2WI, enhancement type on T1WI, uterine anteroposterior diameter, and peripheral blood platelet count can be used as predictors of sufficient ablation.

## Data Availability Statement

The datasets presented in this article are not readily available because the data contains the patient's private information, it cannot be used publicly. Requests to access the datasets should be directed to Jin-Yun Chen, chenjy@cqmu.edu.cn.

## Ethics Statement

The studies involving human participants were reviewed and approved by the Ethics Committee of the First Affiliated Hospital of Chongqing Medical University, and all patients signed informed written consent. The patients/participants provided their written informed consent to participate in this study.

## Author Contributions

M-JY, R-QY, J-YC, W-ZC, and Z-BW conceived and designed the study. M-JY, R-QY, and J-YC accessed and analyzed the data. M-JY, J-YC, and Z-BW developed the methodology. M-JY and J-YC wrote and revised the manuscript. All authors read and approved the final manuscript.

## Conflict of Interest

The authors declare that the research was conducted in the absence of any commercial or financial relationships that could be construed as a potential conflict of interest.
